# Reactive Force Field Development for Propane Dehydrogenation on Platinum Surfaces

**DOI:** 10.1021/acs.jpcc.3c07126

**Published:** 2024-02-09

**Authors:** Antoni Salom-Català, Evgenii Strugovshchikov, Kamila Kaźmierczak, Daniel Curulla-Ferré, Josep M. Ricart, Jorge J. Carbó

**Affiliations:** † Departament de Química Física i Inorgànica, Universitat Rovira i Virgili, 43007 Tarragona, Spain; ‡ TotalEnergies OneTech Belgium, Zone Industrielle Feluy C, 7181 Seneffe, Belgium

## Abstract

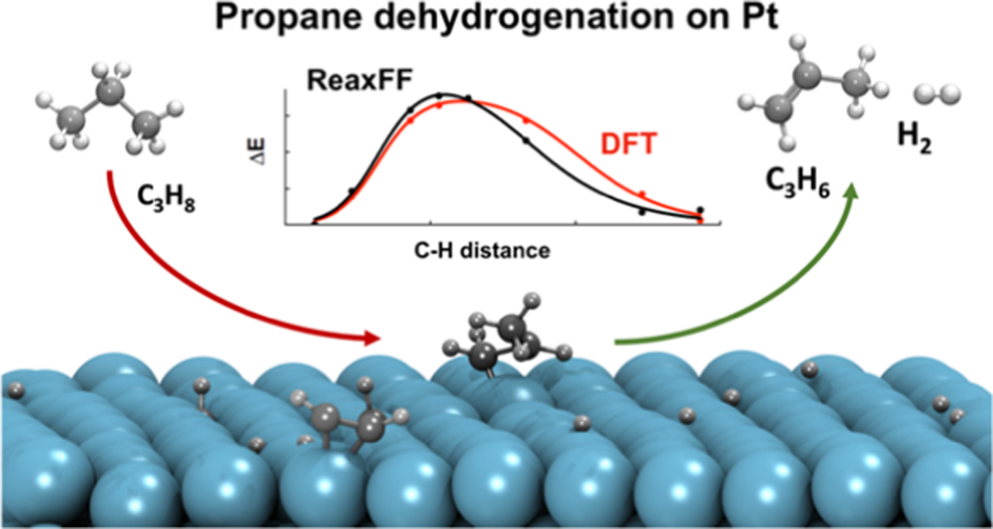

Propane dehydrogenation (PDH) is an on-purpose catalytic technology to produce propylene from propane that operates at high temperatures, 773–973 K. Several key industry players have been active in developing new catalysts and processes with improved carbon footprint and economics, where Pt-based catalysts have played a central role. The optimization of these catalytic systems through computational and atomistic simulations requires large-scale models that account for their reactivity and dynamic properties. To address this challenge, we developed a new reactive ReaxFF force field (**2023-Pt/C/H**) that enables large-scale simulations of PDH reactions catalyzed on Pt surfaces. The optimization of force-field parameters relies on a large training set of density functional theory (DFT) calculations of Pt-catalyzed PDH mechanism, including geometries, adsorption and relative energies of reaction intermediates, and key C–H and C–C bond-breaking/forming reaction steps on the Pt(111) surface. The internal validation supports the accuracy of the developed **2023-Pt/C/H** force-field parameters, resulting in mean absolute errors (MAE) against DFT data of 14 and 12 kJ mol^–1^ for relative energies of intermediates and energy barriers, respectively. We demonstrated the applicability of the **2023-Pt/C/H** force field with reactive molecular dynamics simulations of propane on different Pt surface topologies and temperatures. The simulations successfully model the formation of propene in the gas phase as well as competitive, unproductive reactions such as deep dehydrogenation and C–C bond cleavage that produce H, C_1_ and C_2_ adsorbed species responsible of catalytic deactivation of Pt surface. Results show the following reactivity order: Pt(111) < Pt(100) < Pt(211), and that for the stepped Pt(211) surface, propane activation occurs on low-coordinated Pt atoms at the steps. The measured selectivity as a function of surface topology follows the same trend as activity, the Pt(211) facet being the most selective. The **2023-Pt/C/H** reactive force field can also describe the increase of reactivity with the temperature. From these simulations, we were able to estimate the Arrhenius activation energy, 73 kJ mol^–1^, whose value is close to those reported experimentally for PDH catalyzed by large, supported Pt nanoparticles . The newly developed **2023-Pt/C/H** reactive force field can be used in subsequent investigations of different Pt topologies and of collective effects such as temperature, propane pressure, or H surface coverage.

## Introduction

Over the past few years, the demand for light olefins has increased because of their extensive use as chemical building blocks employed in the production of a vast array of essential chemicals such as polymers, oxygenates, and other important chemical intermediates such as ethylbenzene and propionaldehyde.^1−3^ Among different possible olefins, propene has become one of the most valuable chemical precursors, with a continuously growing demand, used to synthesize polypropylene, acrylonitrile, and propylene oxide.^4^

**Figure 1:**
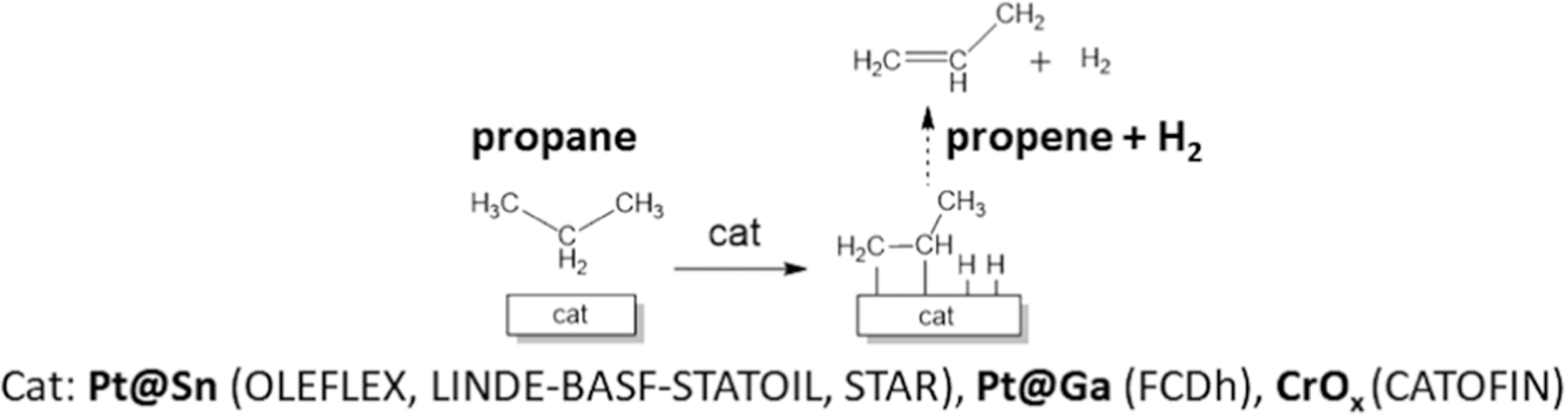
Schematic illustration of the catalytic direct PDH with commercial catalysts used nowadays in the industry.^4^

Typically, light olefins are obtained by steam cracking or fluid catalytic cracking methods of naphtha, light diesel, and other oil byproducts.^5,6^ However, there are limitations to using these methods, such as high-energy demand resulting in a large carbon footprint, low selectivity toward the desired olefin, dwindling petroleum reserves, or rising oil prices. Thus, the use of more efficient conversion methods and technologies is mandatory. Within this context, the catalytic propane dehydrogenation (PDH) is a convenient process for selective propylene production.^7,8^ As shown in Figure 1, this reaction allows obtaining propene and hydrogen directly from propane with no other reagents.^9^ Due to the stable and nonpolar nature of alkanes,^10−12^ the PDH reaction is endothermic (Δ*H*^0^ = +124 kJ mol^–1^ at 298 K), and therefore, it requires high temperatures (773–973 K) and the presence of a catalyst to be commercially feasible. The widely used catalysts for these processes are metal oxides, typically based on chromia (Cr_*x*_O_*y*_), and metals, such as Pt-based compositions.^4,13^ During the last years, the interest in Pt-based catalysts has been caused by the significant health issues derived from possible chromium spilling or exposure, as well as rapid coking and sintering of chromia-based materials.^14−16^ Nevertheless, Pt-based catalysts can undergo rapid deactivation due to coke formation and sintering at high temperatures, which requires implementing regeneration processes.^17^ A possible way to overcome the problem of coke formation is the formulation of new and more efficient catalysts, as well as to optimize reaction conditions, such as temperature, pressure, and gas feed composition.^16^

Several studies provide insight into the PDH reaction at the atomic level within the framework of the DFT modeling.^12,13,18−24^ These studies indicate that the rate-limiting process of PDH by Pt catalysts is the adsorption of propane followed by the first activation of a C–H bond to give adsorbed propyl intermediate.^12,13,20,25^ Calculations have also characterized competitive side reactions such as deep dehydrogenation and C–C bond breaking and compared them with productive propene formation.^23^ Recently, DFT-based kinetic Monte Carlo (KMC) simulations revealed that in the initial stage of PDH, there is a quick deactivation of the active sites in the metal surface, where C_2_/C_1_ carbon species are tightly adsorbed.^12^ These C_2_/C_1_ species formed at the early deactivation stage are proposed to be the precursor of coke formation.^12^ There is no consensus in the bibliography about the topology of the active site of Pt catalyst for PDH. Nevertheless, Fricke et al. have recently combined DFT calculations with Bayesian statistics and reported experimental literature, concluding that Pt(211) is the dominant active facet and is more selective toward propene than Pt(111) and Pt(100).^26^ In the same line, if we compare the energy barriers for the first C–H activation of propane on different Pt surface topologies, we observe the following reactivity trend: Pt(111) < Pt(100) < Pt(211).^23,24^ Also, Zhu et al. have computationally compared Pt(211) and Pt(111) facets, concluding that the activity on Pt(211) is orders of magnitude greater than on Pt(111), while its selectivity depends on the reaction conditions.^27^ Despite all these achievements, the application of DFT methods in the discovery of heterogeneous catalysts in processes such as PDH encounters important issues due to the high degree of complexity of such materials, whose catalytic properties are usually modulated by interfacial effects, particle size, speciation, feed composition, temperature, and pressure. Large-scale models enabling the study of catalytic systems of thousands of atoms and dynamic properties are required to exploit the potential of high-performance computers in the *in silico* discovery of catalytic materials. Current methodologies can hardly address such complexity and require new computational strategies that allow one to operate beyond the atomistic level and with an accuracy close to that of DFT-based methods.

During the last years, reactive Molecular Dynamics (MD) approaches based on force fields, such as ReaxFF,^28−31^ COMB,^32−34^ or AIREBO,^35^ have gained significant attention due to their ability to reproduce chemical reactions, including bond-breaking and bond formation (as *ab initio* Molecular Dynamics, AIMD) for large systems. The ReaxFF developed by van Duin in 2001,^28−31^ uses the so-called bond order (BO) formalism, which is a general relation between bond length and bond order, and further bond order and bond energy, leading to proper bond dissociation/formation energies. The ReaxFF approach has demonstrated significant potential in diverse fields, including investigations of catalyzed reactions,^36−40^ conformational dynamics of biomolecules,^41^ oxidation of metal surfaces,^42^ and other applications.^29,43,44^ Accurate reactive ReaxFF calculations require the assessment of the transferability of existing force-field parameters and, in many cases, the development of specific force-field parameters for the target catalytic process. In the development process, one must generate either computational or experimental reference data for training and validation sets. This process may benefit from the existing DFT literature on the reaction mechanism of PDH catalyzed by Pt,^26^ although often, computational open data do not contain all necessary information, and performing new calculations is necessary.

**Scheme 1:**
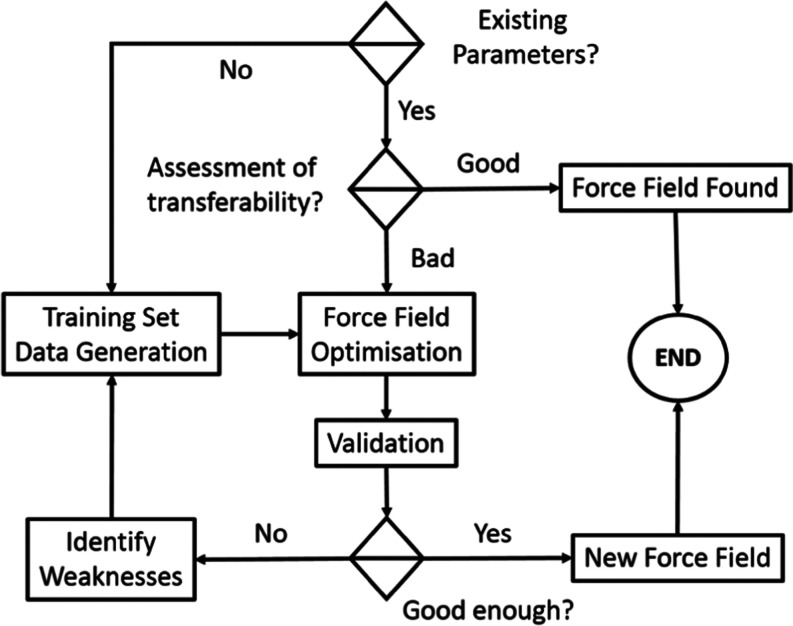
General Workflow for ReaxFF Force-Field Development

This work aims to develop a reactive ReaxFF force field to study the PDH reaction on Pt catalysts. The general procedure for the ReaxFF force-field development is depicted in Scheme 1. Initially, the transferability of existing parameters has to be assessed by comparing them with reference data, for example, a previous computational study on the mechanism of PDH on the Pt(111) surface.^13^ In this case, the force field required reparametrization and generation of a new training set based on DFT calculations on selected reaction intermediates and energy profiles for key C–H and C–C bonds breaking. The commercially available Covariance Matrix Adaptation-Evolution Strategy (CMA-ES) method was employed to optimize the parameter of the ReaxFF force field.

We validated the newly generated ReaxFF force field internally and externally by performing static calculations and reactive dynamic simulations. Moreover, simulations on surfaces of different topologies and temperatures allowed us to gain insight into the PDH mechanism occurring on Pt surfaces.

## Methods

### DFT Calculations

The training data to develop the ReaxFF force-field parameters was generated from density functional theory (DFT) calculations. Vienna ab initio simulation package (VASP 5.3.5)^45−48^ was selected to perform DFT calculations, using the revised-PBE (RPBE-GGA) functional and projector augmented wave (PAW) pseudopotentials.^49,50^ The plane-wave basis set corresponded to 2s^2^2p^2^, 1s^1^, and 5d^9^6s^1^ valence electrons of C, H, and Pt atoms, respectively. The cutoff energy for the plane-waves basis set was set as 400 eV. The Monkhorst–Pack^51^ 3 × 3 × 1 *k*-point mesh was used for the integration in the reciprocal space. The convergence criterion for the electronic minimization was set at 10^–7^ eV, with the forces over all atoms being smaller than 0.03 eV Å^–1^. The partial occupancies of the orbitals were established using the Methfessel-Paxton scheme^52^ with a smearing of 0.2 eV. The Pt(111) surface used in the DFT training set was modeled as a p(3 × 3) 5-layer Pt slab, with a unit cell of 8.44 × 8.44 × 22.17 Å^3^. A data set collection of the structures used to build the training set is available in the ioChem-BD repository^53^ (freely accessible online: 10.19061/iochem-bd-2-66).

### ReaxFF Method

The ReaxFF^28^ interatomic potential was designed to simulate reactive events through a bond order concept. This concept, developed by Abell,^54^ Tersoff,^55,56^ and Brenner,^57^ allows to calculate the bond order empirically at each simulation step from the interatomic distances. Numerically, the ReaxFF potential energy of the system is defined as the sum of different energy contributions:
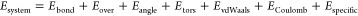
1

The first four terms stand for the covalent contributions and represent the bonding energy between two atoms (*E*_bond_), an energy penalty to prevent the over coordination of atoms (*E*_over_), the three-body angle strain energy (*E*_angle_), and the four-body dihedral strain energy (*E*_tors_). *E*_vdWaals_ and *E*_Coulomb_ are the nonbonded interactions representing the dispersive and electrostatic contributions calculated over all the atoms regardless of connectivity and bond order. The last term (*E*_specific_) is related to some specific energy contributions, calculated only in special cases, such as lone pair energy, conjugation, or hydrogen binding. All of the covalent terms in eq 1 are bond-order-dependent, and since the bond orders are calculated in each step, the bonds can break or form during the simulation. The short-range interactions for the van der Waals energy are described by a distance-corrected Morse potential. The Coulomb energy is calculated by the electronegativity equalization method (EEM).^29,30,33,58,59^

### ReaxFF Calculations

The relative energies of the intermediates and the energy profiles were obtained by static ReaxFF calculations, which have been performed using the parameter estimation L-BFGS algorithm (Limited Memory Broyden–Fletcher–Goldfarb–Shanno algorithm)^60^ with a maximum number of iterations of 10,000 and with a force convergence criterion of 4.2 kJ mol^–1^ Å^–1^. ReaxFF MD simulations were performed at constant temperature (NVT) with a Berendsen thermostat^61^ and a damping constant of 100 fs. The Velocity-Verlet integration method was employed to calculate the trajectories of the particles during MD simulations. The simulation time of MD simulations for productive runs was set to 2 ns, and the time step used was 0.25 fs, which is the default value in the ReaxFF implementation. This was preceded by an initial equilibration at 300 K for 50 ps and heating to working temperature for 50 ps.

### Optimizer of Force-Field Parameters

The force-field parameters were optimized against DFT data using the Covariance Matrix Adaptation Evolution Strategy (CMA-ES)^62−65^ as implemented in the 2020.103 version of the Amsterdam Modeling Suite (AMS) package of programs. Since CMA-ES is a stochastic method, each force-field optimization has been performed 5 times to ensure that the minimization of the error function is close enough to a global minimum. The number of iterations was set to 10,000 for each run with a sample size of 60, bringing the total number of error function evaluations to 600,000.

## Results and Discussion

### Development of Pt/C/H Force-Field Parameters

To obtain ReaxFF Pt/C/H force-field parameters for describing the PDH reaction catalyzed by Pt, we retrained a previous set of atomic and interatomic parameters developed to study the interaction between platinum clusters and carbon platelets.^66^ This force field exhibits an appropriate description of Pt, C, and H atomic parameters, as well as Pt–Pt, Pt–C, and Pt–H interactions and C–H bonding. However, its assessment revealed that these parameters were unable to reproduce the thermodynamics and kinetics of reactive events involving C–H and C–C activations. Note that other ReaxFF Pt/C/H force-field parameters were also developed such as those for describing the adsorption of small C/H molecules (e.g., H, C, CH, CH_2_, CH_3_) on Pt clusters.^67,68^ Still, they are not suitable for describing reactive events in hydrocarbons. Thus, the retraining of Pt/C/H force field^66^ aims to balance the Pt–C/H interactions in physisorbed species (*e.g*., propane) and chemisorbed (e.g., propyl) and to describe C–H, C–C, and H–H bond-breaking/forming processes on the platinum surface. Table S1 lists the retrained force-field parameters.

**Figure 2:**
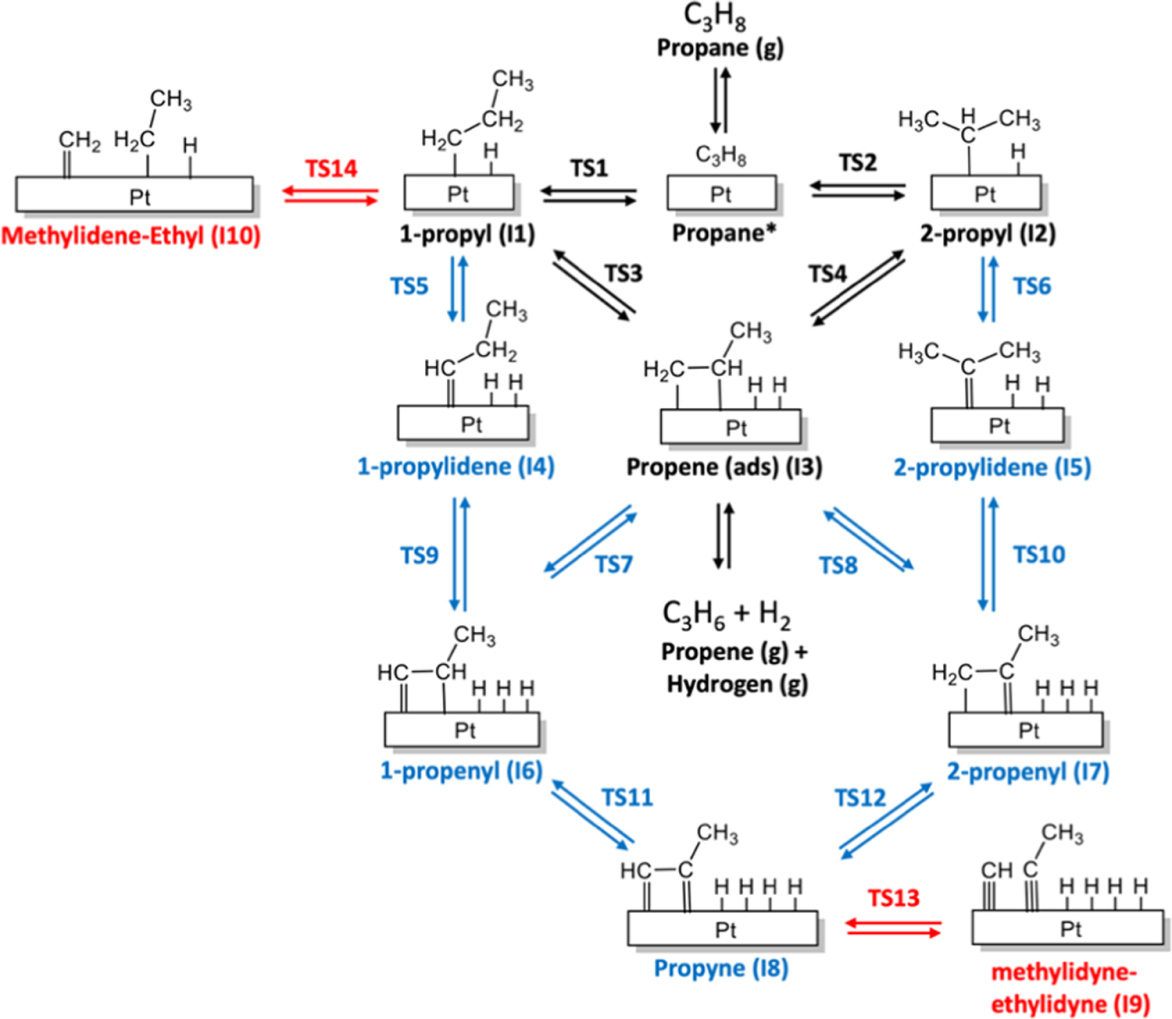
Simplified representation of reaction network for PDH over Pt catalysts proposed by Saerens et al.,^13^ used for building the training set. The main production pathways are colored in black, deep dehydrogenation pathways in blue, and C–C bond-cracking pathways in red.

DFT calculations were used to build a training set, including geometries, adsorption energies of various species, relative energies of reaction intermediates, and energy scans of key elementary steps of the PDH mechanism. As a reference for the training set, we choose the reaction mechanism proposed by Saerens et al.^13^ based on DFT calculations performed on the Pt(111) slab. Figure 2 shows a simplified reaction network during the PDH process over Pt catalysts; the black arrows point out the pathways for propene formation, while the blue and red arrows denote deep dehydrogenation and C–C bond-cracking side reactions, respectively. These later elementary reactions can form different adsorbed intermediates, such as 1-, 2-propenyl, methylidyne, and ethylidyne (see Figure 2), which are considered the precursors of coke formation. Thus, our training set contains the geometries and relative energies of the intermediates depicted in Figure 2, as well as the adsorption energies of propane and propene. For propene, the training set specifically includes the desorption process scan, employing both energy and structure differences derived from DFT calculations. We expect these data to be crucial for ReaxFF simulations because desorption of propene to the gas phase is the entropic driving force for the PDH reaction. Finally, we calculated the energy and the geometry variations of key elementary steps associated with C–H and C–C bond-breaking/forming processes. These scans include first C–H activation at primary and secondary propane carbons on the Pt(111) surface (**TS1** and **TS2** in Figure 2), the corresponding second C–H activation to give the adsorbed propene (**TS3** and **TS4**), further C–H activation at adsorbed propene to give 1-propenyl intermediate (**TS7**, deep dehydrogenation), and of C–C bond-breaking processes at the early and the late stages of the reaction (**TS13** and **TS14** in Figure 2). Note that in the force-field reparameterization, we did not employ the algorithms for transition state search in DFT and ReaxFF, but energy scans of the reaction coordinate. The selection of this procedure is based on two main reasons: (1) the ReaxFF parameters at the beginning of the development are poor and cannot guarantee obtaining the transition state structure;^69^ (2) the energy scans give a smooth energy evolution along the reaction coordinate advance, ensuring in most cases a single energy maximum around the transition state geometry. Table 1 summarizes the training set used in force-field development, which consists of 95 energies, 1236 distances, 3439 angles, and 9595 dihedrals, resulting in a total of 14,365 entries for the fitting. As detailed in the Methods section, the ReaxFF force field was fitted using the CMA-ES method, which has led to the new **2023-Pt/C/H** ReaxFF force field, whose parameters are listed in the Supporting Information.

**Table 1:** Summary of the Training Set Used for the Parametrization of the **Pt/C/H** Force Field

process	specification	entries
relative energies	1-propyl (**I1**), 2-propyl (**I2**), propene (**I3**), 1-propylidene (**I4**), 2-propylidene (**I5**), 1-propenyl (**I6**), 2-propenyl (**I7**), propyne (**I8**), methylidyne ethylidyne (**I9**)	1092
adsorption energy	C_3_H_8_*, C_3_H_6_*	178
desorption process	propene	556
C–H activation	CH_3_CH_2_CH_3_* → CH_3_CH_2_CH_2_* + H* (**TS1**)	5733
	CH_3_CH_2_CH_3_* → CH_3_CHCH_3_* + H* (**TS2**)	
	CH_3_CH_2_CH_2_* + H* → CH_3_CHCH_2_* + 2H* (**TS3**)	
	CH_3_CHCH_3_* + H* → CH_3_CHCH_2_* + 2H* (**TS4**)	
	CH_3_CHCH_2_* + 2H* → CH_3_CHCH* + 3H* (**TS7**)	
C–C activation	CH_3_CCH* + 4H* → CH_3_C* + CH* + 4H* (**TS13**)	6806
	CH_3_CH_2_CH_2_* + H* → CH_3_CH_2_* + CH_2_* + H* (**TS14**)	

### Validation of **2023-Pt/C/H** Force Field

The computed DFT geometries and energies used in the parametrization process were employed to internally validate the **2023-Pt/C/H** force field. The relative energies of PDH reaction intermediates over Pt(111) calculated using the developed force field compare well with the DFT energies (Table S2), giving a mean absolute error (MAE) of 14.2 kJ mol^–1^. ReaxFF portrays the right trend for the adsorption energies of propane and propene that are computed to be isoenergetic and exothermic, respectively (+0.4 kJ mol^–1^ versus −2.9 kJ mol^–1^, and −56.9 kJ mol^–1^ versus −66.5 kJ mol^–1^, for DFT and ReaxFF, respectively). The other chemical trends are also well reproduced by the **2023-Pt/C/H** force field compared to DFT data: (1) the propane C–H bond breaking of the primary carbon (**I1**) is thermodynamically favored over the secondary carbon (**I2**); (2) from the propyl intermediates (**I1** and **I2**), formation of the adsorbed alkene (**I3**) is preferred over the formation of propylidene intermediates (**I4** and **I5**); (3) from adsorbed propene, C_3_H_6_*, deep dehydrogenation is energetically favored with respect to the desorption, but the entropic term at high temperatures can invert this trend; and (4) the adsorbed propyne is more likely to reversely hydrogenate than to further evolve to C–C bond-breaking species.

**Figure 3:**
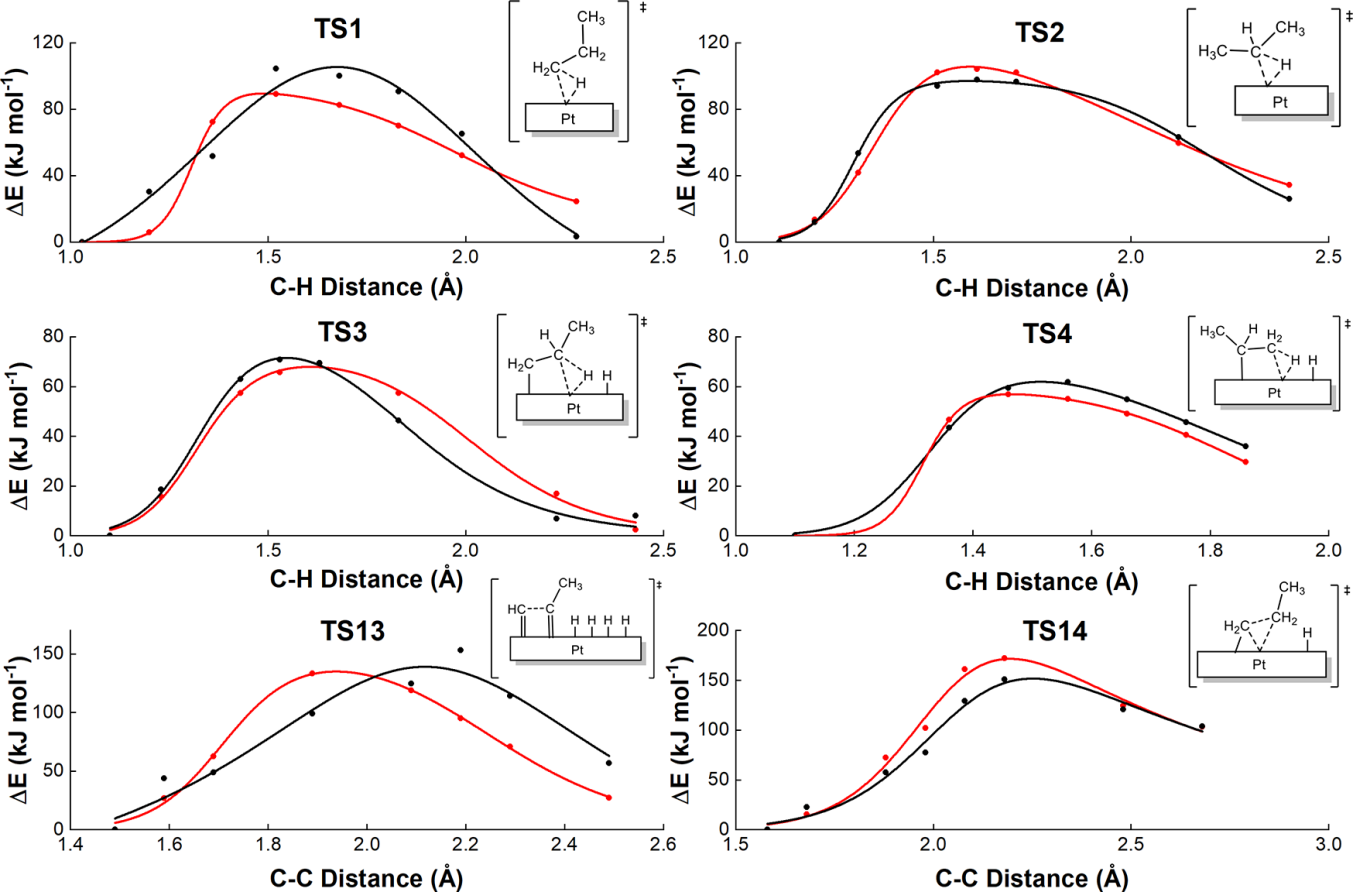
Comparison of ReaxFF and DFT energy scans of four C–H bond activations (**TS1**, **TS2**, **TS3**, and **TS4**) and two C–C bond activations (**TS13** and **TS14**). Black and red lines correspond to ReaxFF and DFT calculations, respectively. Energies are in kJ mol^–1^ and distances in Å.

Figure 3 compares the ReaxFF and DFT results of the energy scan for representative C–H and C–C bond activation over the Pt(111) surface to assess whether the developed force field correctly describes the reaction kinetics. Table S2 lists the estimated energy barriers from these curves derived from ReaxFF and DFT calculations. Although transition state searches can be performed using both ReaxFF and DFT, we fitted these curves to ensure that the ReaxFF energy evolves smoothly with the bond distances; in other words, there are no energy jumps, which could interfere with reaction kinetics. In all cases, the energy and bond distance curves from ReaxFF match well with those from DFT in both value and trend. The MAE for the estimated energy barriers (11.5 kJ mol^–1^) is of the same order as that measured for the relative energies of the reaction intermediates (14.2 kJ mol^–1^, see above).

**Figure 4:**
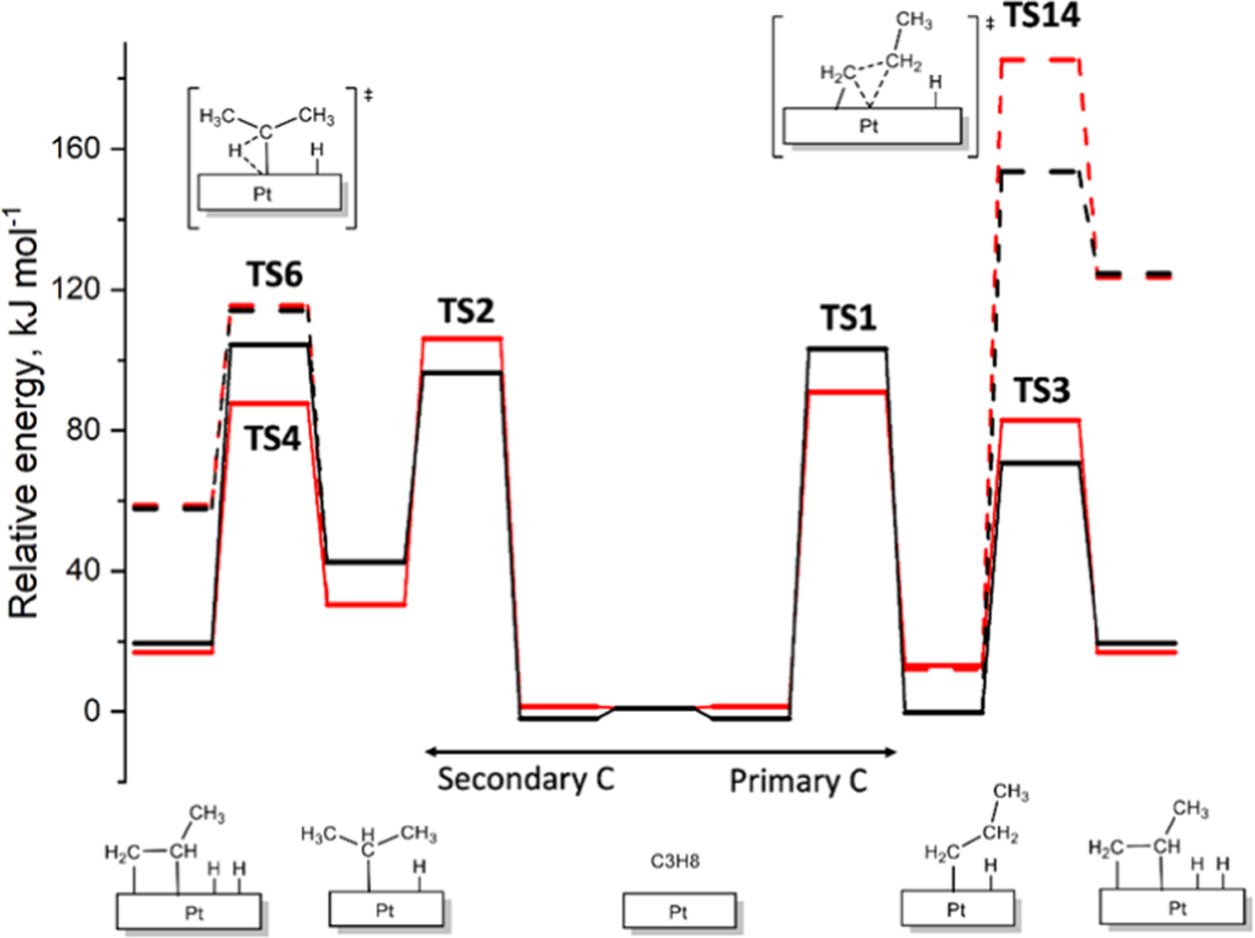
Comparison of ReaxFF and DFT energy profile (kJ mol^–1^) for propane dehydrogenation reaction on Pt(111) surface starting by a C–H activation at primary carbon (right) and at secondary carbon (left). Black and red lines correspond to ReaxFF and DFT calculations, respectively, while dashed lines correspond to side reactions.

As illustrated in Figure 4, which compares the ReaxFF and DFT reaction energy profiles, the **2023-Pt/C/H** force-field parameters reproduce the key trends: (1) the energy barrier for C–C bond breaking is higher than those for C–H activation, and (2) the first C–H bond activations (105 and 98 kJ mol^–1^ for primary (**TS1**) and secondary (**TS2**) carbons, respectively) have higher energy cost than the second C–H bond activation from propyl intermediates (71 and 62 kJ mol^–1^ from 1- (**TS3**) and 2-propyl (**TS4**) intermediates, respectively). This latter trend agrees with previous mechanistic studies indicating that the rate-determining step for the PDH reaction is the first C–H bond activation of propane.^23^ The DFT energy barrier for C–H activation at primary carbons is only slightly lower (15 kJ mol^–1^) than that at secondary carbons, and ReaxFF inverts this trend in a small extent (see Table S3). Nevertheless, reactive MD simulations (see below) show that PDH proceeds preferentially through the primary C–H activation pathway, due to the statistical prevalence of primary C–H bonds over secondary ones (3:1), and the thermodynamically disfavored formation of 2-propyl intermediate resulting from secondary C–H bond activation.

**Figure 5:**
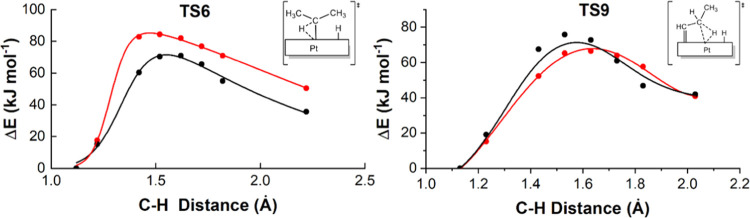
Comparison of ReaxFF and DFT energy scans of two externally validated C–H bond activations (**TS6** and **TS9**). Black and red lines correspond to ReaxFF and DFT calculations, respectively. Energies in kJ mol^–1^ and distances in Å.

We also performed an external validation of **2023-Pt/C/H** force-field parameters by evaluating the energy evolution of two C–H bond activation processes not included in the training set: CH_3_CHCH_3_* + H* → CH_3_CCH_3_* + 2H*, **TS6**; and CH_3_CH_2_CH* + 2H* → CH_3_CHCH* + 3H*, **TS9** (see Figure 2). The ReaxFF calculations reproduce DFT results well (see Figure 5), the absolute errors being 13.4 and 9.2 kJ mol^–1^ for **TS6** and **TS9** elementary reactions, respectively. These errors are similar to those of the MAE (11.5 kJ mol^–1^) determined during internal validation by evaluating the energy barriers of the elementary reaction steps included in the training set.

To further validate externally the **2023-Pt/C/H** force field, we have evaluated the energy barriers for selected C–H bond (**TS1**, **TS2**, **TS3**, and **TS4**) and C–C bond (**TS14**) activations over different types of the platinum surfaces, Pt(111), Pt(100) and Pt(211). Then, we compared them with reported DFT values.^23,24^ Since, in this case, we aim to assess trends rather than values, we compared the variations of the energy barriers on moving from Pt(111) to Pt(100) and Pt(211) surfaces (values reported in Table 2). As a general trend, all the computed energy barriers over Pt(100) and Pt(211), containing low-coordinated Pt atoms, are lower than those on the Pt(111) surface, and this trend is more pronounced for the stepped Pt(211). In line with these trends, the ReaxFF relative energy barriers for the Pt(100) surface are all negative and have even larger negative values for the Pt(211) surface. Thus, the validation of the **2023-Pt/C/H** force field indicates that it is not only able to describe the main features of propane dehydrogenation over Pt(111) but also to gauge the effects of Pt topology on the reaction, enabling the study of different types of Pt catalysts. The ability of the **2023-Pt/C/H** force field to describe the propane reactivity on Pt surfaces with different topologies is further analyzed in the next section.

**Table 2:** Comparison of ReaxFF and DFT Energies of the C–H and C–C Bond-Breaking Processes on Different Pt Topologies^a^

		ΔΔ*E*^‡^	ΔΔ*E*^‡^ (DFT)^b^^,^^c^		ΔΔ*E*^‡^ (ReaxFF)	
elementary reactions		Pt(111)	Pt(100)	Pt(211)	Pt(100)	Pt(211)
CH_3_CH_2_CH_3_* → CH_3_CH_2_CH_2_* + H*	**TS1**	0.0	–25.0	–35.6	–16.3	–26.8
CH_3_CH_2_CH_3_* → CH_3_CHCH_3_* + H*	**TS2**	0.0	–26.8	–40.2	–19.3	–33.0
CH_3_CH_2_CH_2_* → CH_3_CHCH_2_* + H*	**TS3**	0.0	–29.7	–51.0	–15.1	–26.8
CH_3_CHCH_3_* → CH_3_CHCH_2_* + H*	**TS4**	0.0	–28.0	–33.1	–3.3	–28.0
CH_3_CH_2_CH_2_* → CH_3_CH_2_* + CH_2_*	**TS14**	0.0	–50.2	–59.0	–59.4	–65.3

a Energy barriers (kJ mol^–1^) for Pt(100) and Pt(211) are given as relative energies, with Pt(111) used as reference. The negative values denote a lowering of the energy barriers.

b Values taken from ref (23).

c Values taken from ref (24).

### ReaxFF Molecular Dynamic Simulations of Propane Reactivity on the Pt(111), Pt(100), and Pt(211) Surfaces

**Figure 6:**
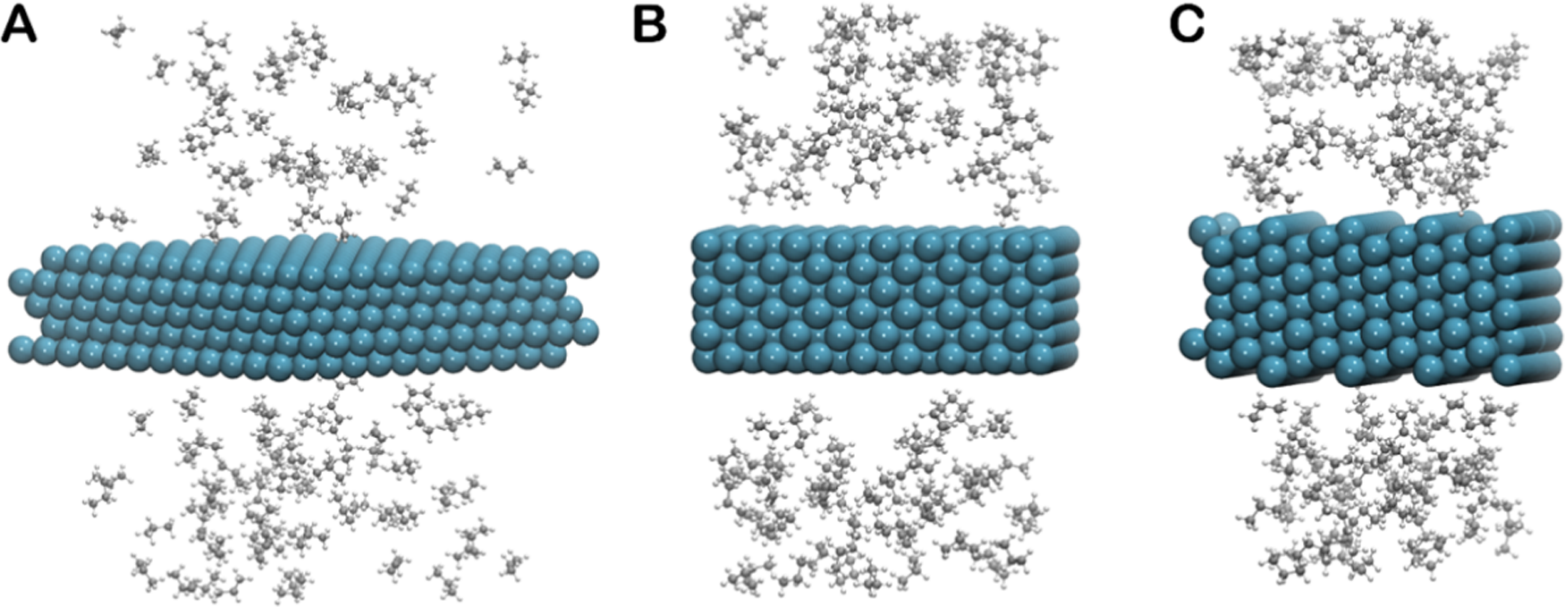
Initial configurations of selected systems to simulate PDH with ReaxFF reactive MD using the **2023-Pt/C/H** force field: (A) Pt(111) surface, (B) Pt(100) surface, (C) Pt(211) surface.

The newly developed ReaxFF **2023-Pt/C/H** force field was used to compare the reactivity of propane on Pt surfaces with different topologies via reactive molecular dynamics (MD) simulations. The selected surfaces are Pt(111), Pt(100), and the stepped Pt(211), and the corresponding simulated systems are shown in Figure 6. The model for the Pt(111) surface was composed of a five-layered p(8 × 8) supercell with box parameters of 29.3 × 33.8 × 51.5 Å^3^, the model for Pt(100) surface was formed by a p(8 × 9) supercell with six layers and a 31.8 × 35.8 × 54.8 Å^3^ box, and the model for Pt(211) surface was composed of a six-layer p(12 × 6) supercell in a box of 45.0 × 27.5 × 51.5 Å^3^. The space between the periodic slabs was 42.0 Å. The dimensions of the Pt models were selected to ensure that the total number of surface Pt atoms is the same for three scenarios (288 for each system), allowing a straightforward comparison of the simulation outcomes. The propane molecules were randomly distributed in the empty space of the simulation box. Initially, we built a system mimicking the experimental working pressures for propane (1–2 bar), but no reaction was observed within the available simulation time (nanoscale). Since our main goal is to evaluate the performance of the **2023-Pt/C/H** force field on different Pt topologies, we increased the number of propane molecules in the simulation box up to 100 to observe reactive events. We ran 5 independent simulations of 2 ns for each, and the results were averaged over the 5 runs. Before the production runs, the systems were equilibrated as described in the Methods section. To compare the different surfaces, the ReaxFF MD simulations were performed at 873 K, and then, the effect of the temperature was studied on the Pt(111) system.

**Figure 7:**
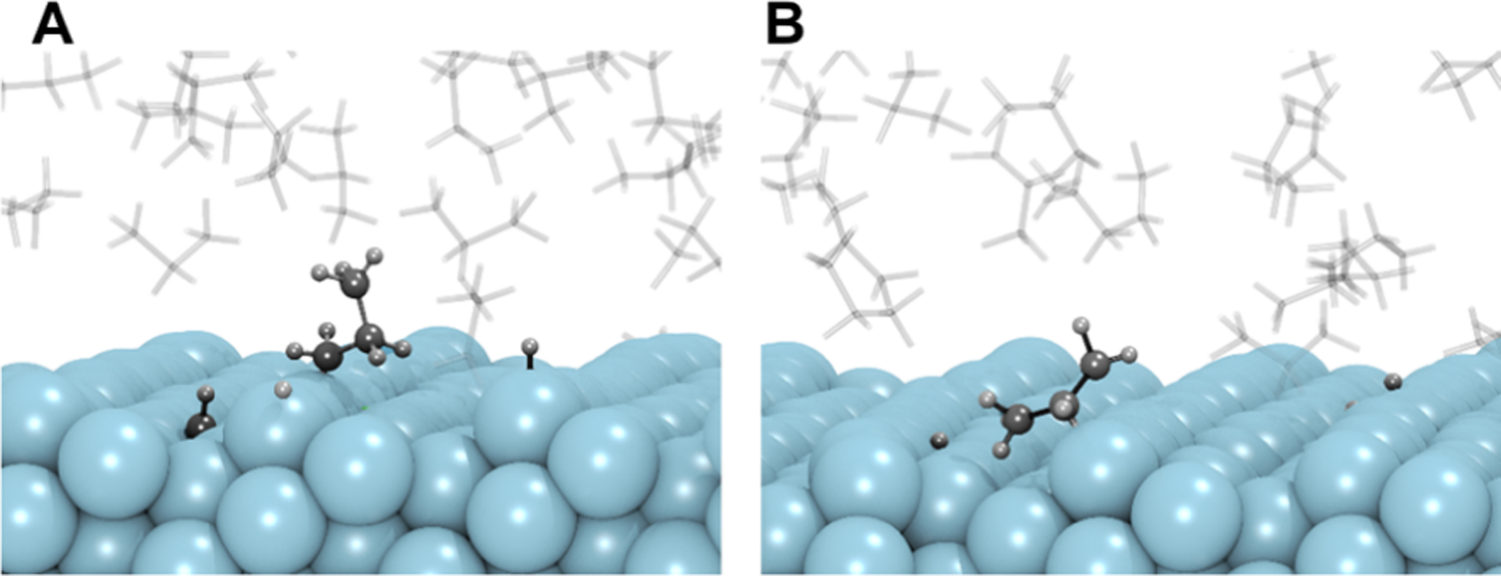
Illustrative snapshots of the formation of 1-propyl (A) and 2-propyl (B) intermediates on the step sites of the Pt(211) surface during the ReaxFF simulation.

Table 3 compares the relative, DFT-computed energy barriers for the initial C–H activation of propane previously reported in the bibliography,^23,24^ with the relative, ReaxFF reaction rates, which are defined as the fraction of reacted propane molecules at the end of the simulation averaged over the five runs and normalized respect to the value for Pt(111) surface. ReaxFF simulations predict the following PDH reactivity order as a function of Pt surface topology: Pt(111) < Pt(100) < Pt(211). This trend correlates with the DFT-computed energy barriers for the initial C–H activation in propane, which has been used as an indicator to evaluate the activity variation on different types of surfaces and catalysts.^19,70−72^ We observed that the reaction is initiated by C–H activation, on either the primary or secondary carbon of propane (**TS1** and **TS2** pathways, respectively, in Figure 2). Then, the 1- or the 2-propyl intermediates undergo a second C–H activation to form the adsorbed propene, which can desorb or further react to give deep dehydrogenated or cracked products. The C–H bond activation and other reactions occur at the step sites for the more reactive stepped Pt(211) surface. This agrees with previous DFT studies, which indicated that step sites play a crucial role in propane activation, explaining the kinetic preference with respect to flat surfaces.^23^Figure 7 depicts representative snapshots of the reactive dynamic simulation of propene formation on the Pt(211) surface. Also, ReaxFF simulations showed that the adsorbed hydrogen atoms, generated from propane dehydrogenation, diffuse on the platinum surfaces, reacting to form H_2_ molecules, which then desorb.

**Table 3:** Comparison of DFT, Relative Energy Barriers for the C–H Activation of Propane (ΔΔE_C–H_^**‡**^, in kJ mol^–1^) with Relative Rates of ReaxFF Reactive MD Simulations of Propane Molecules on Pt(111), Pt(100), and Pt(211) Surfaces^a^

			average product distribution			
surface	ΔΔ*E*_C–H_^‡^ (DFT)^b^	relative rate (ReaxFF MD)	reacted^c^	C_3_ (propene)	C_2_	C_1_
Pt(111)	0.0	1.00	4.2 ± 0.1	1.4 (0.4)	2.8	2.8
Pt(100)	–25.1	1.21	5.2 ± 0.1	1.8 (0.6)	2.2	5.8
Pt(211)	–35.6	1.40	6.0 ± 0.3	1.8 (1.4)	2.8	7.0

a Average product distribution (reacted propane molecules and C_3_, C_2_, and C_1_ hydrocarbon species) after five simulation runs of 2 ns each.

b Values taken from refs (23) and (24).

c Uncertainty estimation by comparing the average values derived from the two largest ensembles, *N* = 4 and *N* = 5 simulation runs.

**Figure 8:**
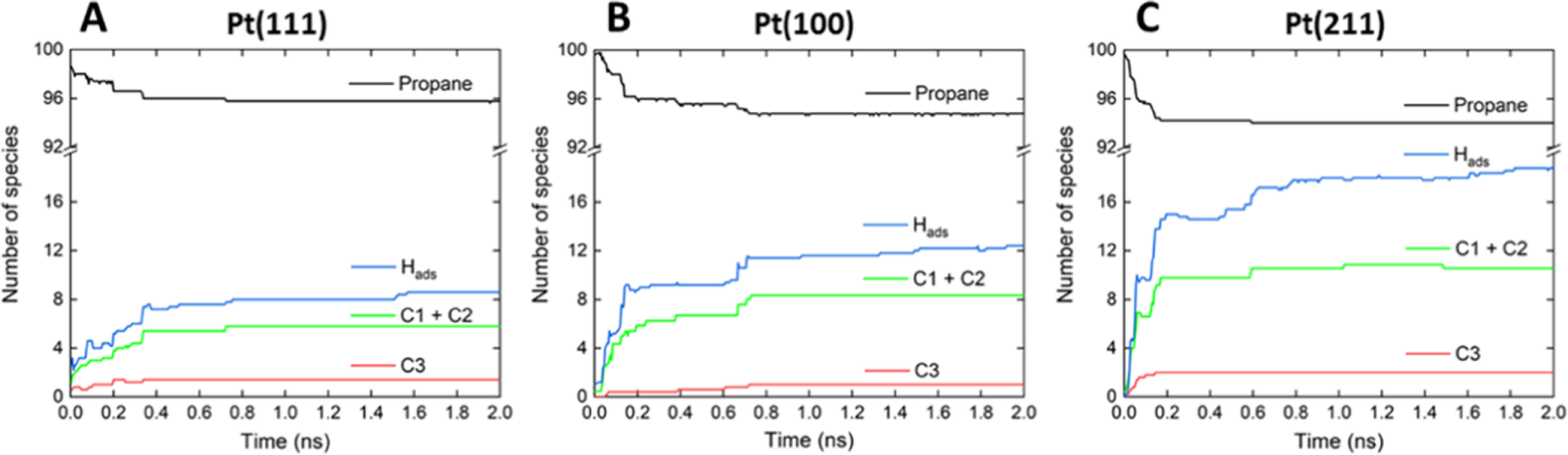
Evolution of the average number of species during the 5 simulation runs of 2 ns for systems defined in Figure 6, Pt(111) facet (A), Pt(100) facet (B), and Pt(211) facet (C).

**Figure 9:**
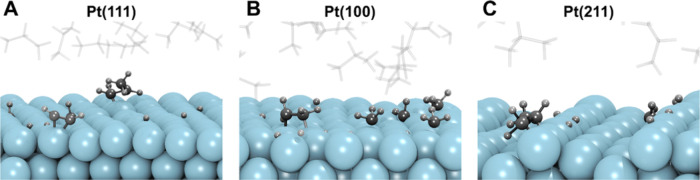
Illustrative snapshots of the formation of deep dehydrogenated and cracked species on Pt(111) (A), Pt(100) (B), and Pt(211) (C) surfaces during ReaxFF simulations.

Figure 8 compares the evolution of different species during the propane dehydrogenation simulation on the three different platinum surface models considered. Interestingly, for all of the surfaces, the propane conversion rate drops significantly before the end of the simulation. We theorize that platinum site occupancies become relevant as the percentage of free sites decreases. In other words, the deep dehydrogenation and the cracking generate H, C_1_, and C_2_ hydrocarbon, adsorbed species that deactivate the platinum surface, blocking the reaction of further propane molecules. In addition, these C_1_ and C_2_ species can be seen as precursors of observed coke formation. This is in line with recent kinetic Monte Carlo simulations, which indicated that in the initial stages of the reaction, there is a quick deactivation in the metal surface, where C_1_ and C_2_ hydrocarbon species are strongly adsorbed.^12^ In our simulations, the fast formation of coke precursors is likely to result from the high density of propane reactant molecules required to observe reactive events within the available times. Figure 9 shows representative snapshots of the ReaxFF simulations illustrating the adsorption of hydrogen and hydrocarbons on different surfaces. We observed the formation of different deep dehydrogenated C_3_ species such as allyl*, 1-propenyl*, or propyne*, and also of other cracked C_2_ and C_1_ species such as C_2_H_5_*, C_2_H_4_*, and CH_3_*. The reactivity order for deep dehydrogenation and cracking as a function of Pt surface topology follows the same trend as the PDH reaction: Pt(111) < Pt(100) < Pt(211); see Table 3 and Figure 8. At the end of our simulations, we estimated that the percentages of Pt atoms on the surface affected by adsorbed species (first and second interaction spheres) are 26.2, 36.6, and 49.4% for Pt(111), Pt(100), and Pt(211), respectively. The selectivity to propene for the different surfaces can be estimated as the ratio between the number of formed propene molecules and the number of propane molecules transformed into C_2_ and C_1_ species via cracking and deep dehydrogenation. In these simulations, the C_3_ deep dehydrogenated species are statistically irrelevant. From the data in Table 3, we find that Pt(211) is the most selective of the surfaces, followed by Pt(100) and Pt(111). The calculated selectivities are relatively low (ranging from 13 to 25%), probably due to the artificially high propane concentration required for our simulations. Nevertheless, we were able to predict the Pt(211) facet as the more selective, in agreement with a recent study, in which six kinetic models were validated using data from different DFT functionals and experiments.^26^

**Figure 10:**
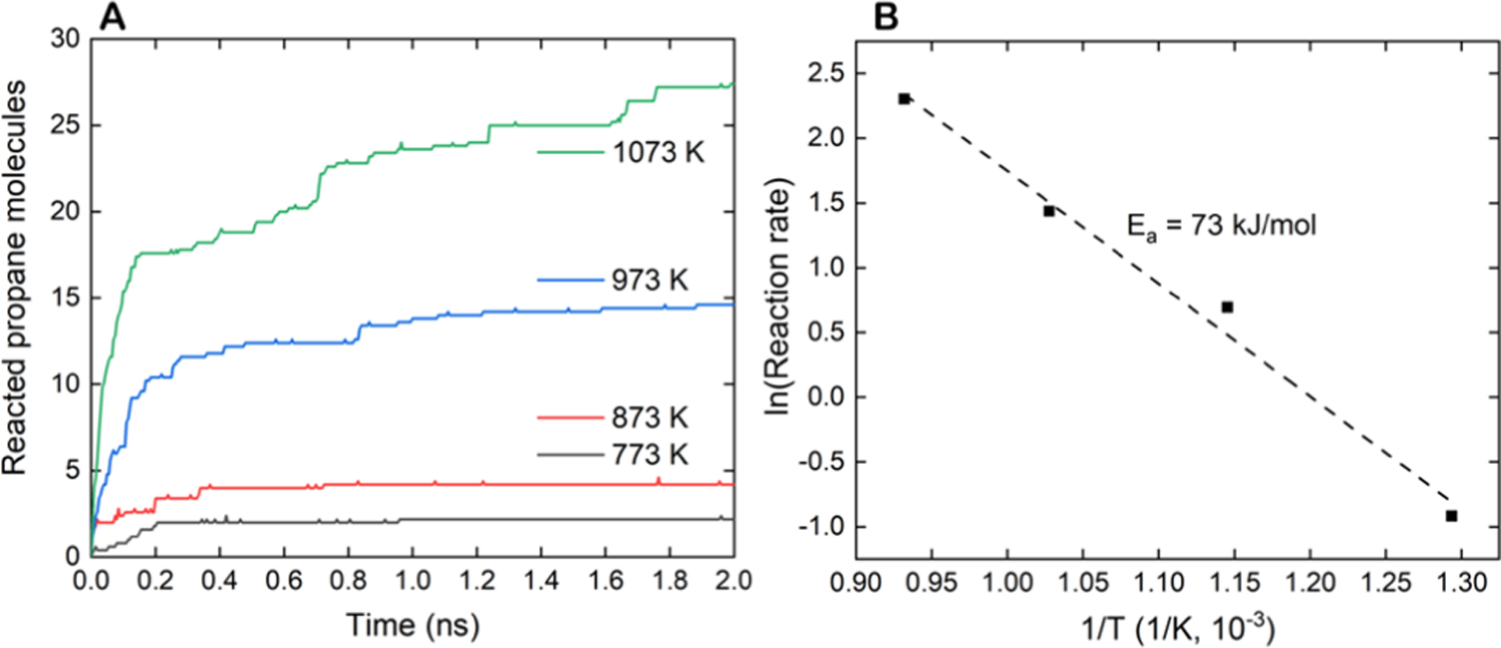
Evolution of the average number of species during the five simulation runs of 2 ns on the Pt(111) facet at different temperatures (A) and the Arrhenius plot derived from simulations (B).

To evaluate the ability of the ReaxFF force field to reproduce the temperature-dependent behavior of the Pt catalyst, simulations of propane dehydrogenation were also conducted on a Pt(111) surface at different temperatures (773, 873, 973, and 1073 K). The results of the simulations are summarized in Table 4 and Figure 10. Propane conversion increases exponentially with increasing temperature and, similarly, the generation of C_2_ and C_1_ hydrocarbon species adsorbed on the surface. These findings indicate that, although higher temperatures may lead to more efficient propane conversion, it may also result in a more significant amount of coke formation on the platinum surface, subsequently leading to the deactivation of the catalyst.^17^

**Table 4:** ReaxFF Reactive MD Simulations of Propane Molecules on the Pt(111) Surface at Different Temperatures^a^

		average product distribution			
*T*, K	relative rate (ReaxFF MD)	reacted	C_3_ (propene)	C_2_	C_1_
773	1.0	2.2	1.1 (0.0)	1.0	1.0
873	3.3	4.2	1.4 (0.4)	2.8	2.8
973	8.3	14.8	4.2 (1.4)	9.8	11.6
1073	19.3	27.6	8.2 (2.6)	17.6	23.0

a Relative rate and average product distribution of reacted propane molecules (C_3_, C_2_, and C_1_ hydrocarbon species) after 5 simulation runs of 2 ns each.

Reactive simulations at different temperatures can also be used to determine the apparent activation energy of the reaction and compare it with experimental values.^73^ To build the Arrhenius plot (Figure 10B), we estimated the rate constants at initial times to avoid the effect of excessive deactivation of the platinum surface due to the high density of propane used in simulations (see discussion above). The concentration of reacted propane was replaced by the number of reacted propane molecules, and the same time interval was considered at each temperature to obtain the initial reaction rate. Initially, we selected the minimal reaction time satisfying the linearity requirements of the Arrhenius plot, the natural logarithm of the reaction rate against the inverse temperature. Using as threshold *r*^2^ ≥ 0.99, the simulation time of 0.045 ns was initially chosen to build the Arrhenius plot (see Figure S1), providing an apparent activation energy (*E*_a_) of 73 kJ mol^–1^. The computed average value of *E*_*a*_ along the time interval from 0.045 to 0.120 ns, where all points exhibit linear plots (*r*^2^ ≥ 0.99), is 68 kJ mol^–1^ with a standard deviation of 3 kJ mol^–1^ (see Table S4). These estimates are in good agreement with the upper value reported for PDH over Pt/Al_2_O_3_ nanocatalysts, in which the apparent activation energy increases with the size of the Pt catalyst until it reaches 75 kJ mol^–1^ for large nanoparticles of ∼5 nm.^74,75^

## Conclusions

In the present work, we have developed the **2023-Pt/C/H** ReaxFF reactive force field for simulating propane dehydrogenation (PDH) on Pt surfaces. The parametrization was performed using a DFT-based training set, including geometries, adsorption and relative energies of the reaction intermediates on Pt(111), and key elementary reaction steps of selected C–H and C–C bond-breaking processes. There is overall good agreement between ReaxFF and DFT results in the internal validation, with mean absolute errors (MAEs) for relative energies of intermediates and energy barriers of 14 and 12 kJ mol^–1^, respectively. The external validation revealed that the **2023-Pt/C/H** force field not only describes the main reaction features of PDH on the Pt(111) surface but also can gauge the effects of Pt topology on the reaction, enabling the study of different types of Pt catalysts.

To further test the developed force field and get insight into the reactivity of propane on Pt surfaces with different topologies, we performed reactive MD simulations on Pt(111), Pt(100), and Pt(211) surfaces and also at different temperatures for Pt(111). ReaxFF simulations on large-scale systems at experimental temperatures predict the following reactivity order: Pt(111) < Pt(100) < Pt(211). In the most reactive Pt(211) surface, the propane activation is exclusively observed on low-coordinated Pt atoms at the steps, which are crucial to enhance catalytic activity. Simulations show quick deactivation in the platinum surface, where H, C_1_, and C_2_ hydrocarbon species, formed from deep dehydrogenation and the C–C cracking side reactions, are strongly adsorbed. These C_1_ and C_2_ species can be precursors of the experimentally observed coke formation, poisoning the catalyst and reducing selectivity toward propene formation. The measured selectivity as a function of surface topology follows the order Pt(211) > Pt(100) > Pt(111). Simulations at different temperatures on the Pt(111) surface allowed building Arrhenius plots to determine computationally the apparent activation energy, 73 kJ mol^–1^, which is close to that determined experimentally for large nanoparticles (∼5 nm) supported on alumina (75 kJ mol^–1^).

All in all, the **2023-Pt/C/H** ReaxFF reactive force field can be employed to study propane dehydrogenation and its side reactions on different Pt catalysts, including simulations of collective effects such as temperature, propane pressure, and H surface coverage. This will enable analyzing more complex Pt systems under different conditions, driving research toward designing more efficient catalytic processes.

## Supporting Information Available

Additional details of force-field development and validation, **2023-Pt/C/H** ReaxFF reactive force-field parameters, DFT data of the training set through the open-access repository ioChem-BD, and the files “geo” and “trainingset” used as input in AMS optimization of ReaxFF parameters (PDF)
Information on the training set that can be used to re-train the ReaxFF force field (TXT)
Information on the geo set that can be used to re-train the ReaxFF force field (TXT)
