# Supplementary material for: Reactive Force Field Development for Propane Dehydrogenation on Platinum Surfaces
Source: J Phys Chem C Nanomater Interfaces. 2024 Feb 9;128(7):2844–55. doi: 10.1021/acs.jpcc.3c07126 (PMC10895921; doi:10.1021/acs.jpcc.3c07126)
Supplement: Supplementary file 1 — jp3c07126_si_001.pdf [file jp3c07126_si_001.pdf]

Supporting information for:

Reactive Force Field Development for Propane Dehydrogenation on Platinum Surfaces

Antoni Salom-Català,[†,§] Evgenii Strugovshchikov,[†,§] Kamila Kaźmierczak,[‡] Daniel Curulla-Ferré,[‡*] Josep M. Ricart, [†*] Jorge J. Carbó[†*]

[†]Departament de Química Física i Inorgànica, Universitat Rovira i Virgili, 43007 Tarragona, Spain

[‡]TotalEnergies OneTech Belgium, Zone Industrielle Feluy C, 7181 Seneffe, Belgium

[§] These two authors equally contributed to this work

Table of contents

Details of ReaxFF force field development procedure, and application S2

ReaxFF **2023-Pt/C/H** force field parameters S5

Details of ReaxFF force field, development procedure, validation, and application

Table S1. List of the reoptimized parameters for **2023-Pt/C/H** force-field

Type	Set of parameters
**Bond**	C-C, C-H, C-Pt
**Off-diagonal**	C-H, C-Pt, H-Pt
**Valence angle**	C-C-C, C-C-H, H-C-H, C-C-Pt, Pt-C-Pt, C-Pt-Pt, C-Pt-C, H-C-Pt, Pt-H-Pt, H-Pt-Pt,
**Torsion terms**	C-C-C-H, H-C-C-H

Table S2. Comparison of the selected PDH reaction steps calculated using DFT and **2023-Pt/C/H** force field. Energies in kJ mol^{-1}. AE = Absolute Error.

Reaction	DFT	2023-Pt/C/H	AE
$CH_3CH_2CH_3(g) \rightarrow CH_3CH_2CH_3*$	+0.4	-2.9	3.4
$CH_3CH_2CH_3(g) \rightarrow CH_3CH_2CH_2* + H*$	+12.2	-1.3	13.4
$CH_3CH_2CH_3(g) \rightarrow CH_3CHCH_3* + H*$	+29.4	+41.6	12.2
$CH_3CH_2CH_2* + H* \rightarrow CH_3CHCH_2* + 2H*$	+3.8	+19.7	16
$CH_3CH_2CH_2* + H* \rightarrow CH_3CH_2CH* + 2H*$	+23.9	+29.8	5.9
$CH_3CH_2CH_2* + H* \rightarrow CH_3CH_2* + CH_2* + H*$	+111.3	+124.7	13.4
$CH_3CHCH_3* + H* \rightarrow CH_3CHCH_2* + 2H*$	-13.4	-23.1	9.2
$CH_3CHCH_3* + H* \rightarrow CH_3CCH_3* + 2H*$	+28.1	+15.1	13
$CH_3CHCH_2* \rightarrow CH_3CHCH_2 (g)$	+57.1	+66.8	9.7
$CH_3CHCH_2* \rightarrow CH_3CHCH* + H*$	+22.3	+21.8	0.4
$CH_3CHCH_2* \rightarrow CH_3CCH_2* + H*$	+16.4	+50.0	33.6
$CH_3CH_2CH* + 2H* \rightarrow CH_3CHCH* + 3H*$	+2.1	+11.8	9.7
$CH_3CCH_3* + 2H* \rightarrow CH_3CCH_2* + 3H*$	-25.2	+12.2	37.4
$CH_3CHCH* + 3H* \rightarrow CH_3CCH* + 4H*$	+18.1	+21.8	3.8
$CH_3CCH_2* + 3H* \rightarrow CH_3CCH* + 4H*$	+23.9	-6.7	30.7
$CH_3CCH* + 4H* \rightarrow CH_3C* + CH* + 4H*$	-5.9	+12.2	18.1

Table S3. Comparison of the estimated energy barriers for C−H and C−C bond breaking processes calculated using DFT and **2023-Pt/C/H** force field. Energies barriers in kJ mol^{-1}. AE = Absolute Error.

**Reaction**	**DFT**	**2023-Pt/C/H**	**AE**
$CH_3CH_2CH_3$* → $CH_3CH_2CH_2$* + H*	89.5	105.0	15.5
$CH_3CH_2CH_3$* → CH_3CHCH_3* + H*	104.6	98.3	6.3
$CH_3CH_2CH_2$* + H* → CH_3CHCH_2* + 2H*	69.7	71.0	1.3
CH_3CHCH_3* + H* → CH_3CHCH_2* + 2H*	57.1	61.7	4.6
CH_3CHCH_3* + H* → CH_3CCH_3* + 2H*	84.8	71.4	13.4
CH_3CH_2CH* + 2H* → CH_3CHCH* + 3H*	66.8	76.0	9.2
CH_3CCH* + 4H* → CH_3C* + CH* + 4H*	133.6	153.7	20.2
$CH_3CH_2CH_2$* + H* → CH_3CH_2* + CH_2* + H*	173.0	151.6	21.4

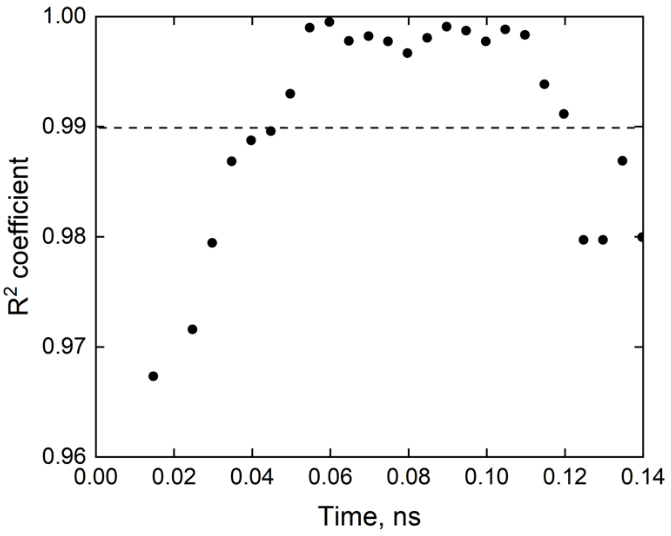

Figure S1. Time evolution of the coefficient of determination (r^2) of the Arrhenius plots derived from 5 simulation runs on the Pt(111) facet at different temperatures. The first 0.14 ns were selected.

Table S4. Activation energy values derived from Arrhenius plots built at different initial times using the average data of 5 simulation runs on the Pt(111) surface at different temperatures. We selected the values in which the correlation coefficient (r^2) is equal to or greater than 0.99.

**Time**, ns	**Activation energy**, kJ mol^{-1}	r^2
0.045	73.4	0.9900
0.050	75.3	0.9929
0.055	67.0	0.9989
0.060	68.6	0.9995
0.065	69.2	0.9977
0.070	71.0	0.9982
0.075	70.1	0.9977
0.080	65.3	0.9966
0.085	64.9	0.9980
0.090	66.2	0.9990
0.095	66.8	0.9987
0.100	67.8	0.9977
0.105	67.4	0.9988
0.110	69.2	0.9983
0.115	65.0	0.9938
0.120	65.6	0.9911
**Average value**	**68.3**	
**Standard deviation**	**3.0**	

ReaxFF 2023-Pt/C/H force field parameters.

```
39       ! Number of general parameters
  50.0000 !p_boc1 Eq(4c): Overcoordination parameter
   9.5469 !p_boc2 Eq(4d): Overcoordination parameter
  26.5405 !p_coa2 Eq(15): Valency angle conjugation
   4.6420 !p_trip4 Eq(20): Triple bond stabilisation
   7.2181 !p_trip3 Eq(20): Triple bond stabilisation
   0.0000 !k_c2 Eq(19): C2-correction
   1.0588 !p_ovun6 Eq(12): Undercoordination
   9.0000 !p_trip2 Eq(20): Triple bond stabilisation
  11.2373 !p_ovun7 Eq(12): Undercoordination
  13.3056 !p_ovun8 Eq(12): Undercoordination
 -15.0000 !p_trip1 Eq(20): Triple bond stabilization
   0.0000 !Lower Taper-radius (must be 0)
  10.0000 !R_cut Eq(21): Upper Taper-radius
   2.8793 !p_fe1 Eq(6a): Fe dimer correction
  33.8667 !p_val6 Eq(13c): Valency undercoordination
   6.0891 !p_lp1 Eq(8): Lone pair param
   1.0563 !p_val9 Eq(13f): Valency angle exponent
   2.0384 !p_val10 Eq(13g): Valency angle parameter
   6.1431 !p_fe2 Eq(6a): Fe dimer correction
   6.9290 !p_pen2 Eq(14a): Double bond/angle param
   0.3989 !p_pen3 Eq(14a): Double bond/angle param
   3.9954 !p_pen4 Eq(14a): Double bond/angle param
  -2.4837 !p_fe3 Eq(6a): Fe dimer correction
   5.7796 !p_tor2 Eq(16b): Torsion/BO parameter
  10.0000 !p_tor3 Eq(16c): Torsion overcoordination
   1.9487 !p_tor4 Eq(16c): Torsion overcoordination
  -1.2327 !p_elho Eq(26a): electron-hole interaction
   2.1645 !p_cot2 Eq(17b): Conjugation if tors13=0
   1.5591 !p_vdW1 Eq(23b): vdWaals shielding
   0.1000 !Cutoff for bond order (*100)
   2.1365 !p_coa4 Eq(15): Valency angle conjugation
   0.6991 !p_ovun4 Eq(11b): Over/Undercoordination
```

```
   1.2593 !p_ovun3 Eq(11b): Over/Undercoordination
   1.8512 !p_val8 Eq(13d): Valency/lone pair param
   0.5000 !X_soft Eq(25): ACKS2 softness for X_ij
  20.0000 !d Eq(23d): Scale factor in lg-dispersion
   5.0000 !p_val Eq(27): Gauss exponent for electrons
   0.0000 !1 Eq(13e): disable undecoord in val angle
   2.6962 !p_coa3 Eq(15): Valency angle conjugation
 4    ! Nr of atoms; cov.r; valency;a.m;Rvdw;Evdw;gammaEEM;cov.r2;#
            alfa;gammavdW;valency;Eunder;Eover;chiEEM;etaEEM;n.u.
            cov r3;Elp;Heat inc.;bo131;bo132;bo133;softcut;n.u.
            ov/un;val1;n.u.;val3,vval4
C    1.3644   4.0000  12.0000   1.9803   0.1720   0.8712   1.2395   4.0000
     9.4734   2.1241   4.0000  31.8793  79.5548   5.7254   6.9235   0.0000
     1.2636   0.0000  -0.0537   5.7133  33.5629  11.9957   0.8563   0.0000
    -2.8983   4.7820   1.0564   4.0000   2.9663   0.0000   0.0000   0.0000
H    0.6853   1.0000   1.0080   1.3588   0.0622   0.9895  -0.1000   1.0000
     9.3992   5.0518   1.0000   0.0000 121.1250   5.7873   7.4100   1.0000
    -0.1000   0.0000  -0.1609   2.5817   4.1491   1.2385   1.0698   0.0000
   -15.7683   2.1488   1.0338   1.0000   2.8793   0.0000   0.0000   0.0000
Pt   1.9318   2.0000 195.0800   2.0163   0.2920   0.6396  -1.0000   2.0000
    12.3885   6.1726   2.0000   0.0000   0.0000   6.3388   5.7617   0.0000
    -1.0000   0.0000 143.1770  22.5697   7.4147   0.1552   0.8563   0.0000
    -7.3154   1.8009   1.0338   5.0000   2.5791   0.0000   0.0000   0.0000
X   -0.1000   2.0000   1.0080   2.0000   0.0000   1.0000  -0.1000   6.0000
    10.0000   2.5000   4.0000   0.0000   0.0000   8.5000   1.5000   0.0000
    -0.1000   0.0000  -2.3700   8.7410  13.3640   0.6690   0.9745   0.0000
   -11.0000   2.7466   1.0338   6.2998   2.8793   0.0000   0.0000   0.0000
 6    ! Nr of bonds; Edis1;LPpen;n.u.;pbe1;pbo5;13corr;pbo6
                       pbe2;pbo3;pbo4;n.u.;pbo1;pbo2;ovcorr
 1  1 147.9403 113.3033  56.7512   0.4407  -0.8510   1.0000  69.3864   0.1509
         0.0040  -0.2802   8.1303   1.0000  -0.1046   6.5354   1.0000   0.0000
 1  2 152.9122   0.0000   0.0000  -0.5495   0.0000   1.0000   6.0000   0.5795
        17.7111   1.0000   0.0000   1.0000  -0.0178   6.7615   0.0000   0.0000
 2  2 167.4522   0.0000   0.0000  -0.3573   0.0000   1.0000   6.0000   0.7489
         9.6471   1.0000   0.0000   1.0000  -0.0169   5.9140   0.0000   0.0000
```

1 3 125.3745 0.0000 0.0000 0.0936 -0.2000 1.0000 16.0000 0.4170
0.0037 -0.2000 15.0000 1.0000 -0.1277 6.9047 1.0000 0.0000
2 3 159.6185 0.0000 0.0000 -0.2169 0.0000 1.0000 6.0000 0.3031
17.4869 1.0000 0.0000 1.0000 -0.0809 10.6932 0.0000 0.0000
3 3 122.1396 0.0000 0.0000 -0.2768 -0.2000 0.0000 16.0000 0.2903
0.7541 -0.2000 15.0000 1.0000 -0.0931 4.7088 0.0000 0.0000
3 ! Nr of off-diagonal terms; Ediss;Ro;gamma;rsigma;rpi;rpi2
1 2 0.0531 1.4051 10.4758 1.1000 -1.0000 -1.0000
1 3 0.0731 1.5105 12.9663 1.7700 -1.0000 -1.0000
2 3 0.0262 2.0620 11.6031 1.6031 -1.0000 -1.0000
17 ! Nr of angles;at1;at2;at3;Thetao,o;ka;kb;pv1;pv2
1 1 1 77.0506 29.3757 0.9103 0.0000 2.9974 1.3978 1.0400
1 1 2 69.0044 12.4410 2.2176 0.0000 0.0028 0.0000 1.0400
2 1 2 85.9036 18.9333 4.3331 0.0000 9.9925 0.0000 1.0400
1 2 2 0.0000 0.0000 6.0000 0.0000 0.0000 0.0000 1.0400
1 2 1 0.0000 3.4110 7.7350 0.0000 0.0000 0.0000 1.0400
2 2 2 0.0000 27.9213 5.8635 0.0000 0.0000 0.0000 1.0400
1 1 3 48.4971 6.4160 7.6245 0.0000 0.1406 0.0000 2.3133
3 1 3 62.6498 17.9545 6.0292 0.0000 0.1002 0.0000 1.0587
1 3 3 50.7074 1.0000 0.5505 0.0000 0.7199 0.0000 2.5983
1 3 1 37.7278 7.5385 2.2595 0.0000 1.0519 0.0000 1.9959
2 1 3 36.0961 12.2768 2.9068 0.0000 0.3731 0.0000 3.0000
1 3 2 86.0878 1.3988 0.2231 0.5000 1.3748 0.0000 1.5790
1 2 3 0.0000 2.3184 0.5764 0.0000 0.1752 0.0000 1.0618
2 3 2 57.3916 22.7625 0.8385 0.0000 1.9462 0.0000 1.1000
2 2 3 0.0000 3.3503 1.6083 0.0000 0.3066 0.0000 1.5060
3 2 3 0.0000 3.2641 2.7545 0.0000 0.9173 0.0000 3.4080
2 3 3 179.9795 -90.6363 3.2076 0.0000 5.1749 0.0000 2.5754
6 ! Nr of torsions;at1;at2;at3;at4;;V1;V2;V3;V2(BO);vconj;n.u;n
1 1 1 1 0.0000 38.9174 0.3649 -8.2931 -2.0127 0.0000 0.0000
1 1 1 2 0.0000 42.4487 0.3409 -8.8361 -1.6776 0.0000 0.0000
2 1 1 2 0.0000 10.5414 2.7558 -5.9189 -6.7268 0.0000 0.0000
0 1 2 0 0.0000 0.0000 0.0000 0.0000 0.0000 0.0000 0.0000
0 2 2 0 0.0000 0.0000 0.0000 0.0000 0.0000 0.0000 0.0000
0 1 1 0 0.0000 0.6675 0.0000 -8.2352 0.0000 0.0000 0.0000

0 ! Nr of hydrogen bonds;at1;at2;at3;Rhb;Dehb;vhb1
